# Mr. Hunter, on Cerussa Acetata

**Published:** 1803-02-01

**Authors:** Alexander Hunter

**Affiliations:** Dumbarton


### *To the Editors of the Medical and Physical Journal.*

Gentlemen,

In Vol. vii. Numb. xl. page 507, of your Journal, is a paper by Mr. Ryan, of Kilkenny, on the use of Acetite of Lead, in cases of Hæmoptysis, from which it appears to have had very beneficial effects; and he is anxious to show that no ill consequences took place, although given in much larger doses than are commonly prescribed.

If the following facts can have any tendency to bring into practice a useful medicine, you may think them worth a place in your useful Miscellany.

An apprentice of William Ewing, a cooper, in this neighbourhood, had an ulcer on the fore part of the tibia with considerable inflammation, for which he was ordered a poultice with acetite of lead. As this article is used by linen-printers, he got from an adjoining print-field, a lump which could not have weighed less than a pound, being, as he declared, the size of his fist, which was none of the smallest.

When the boy came home, he laid down this piece on the kitchen table, and left it. Shortly after his mistress, an old, short-sighted woman, came in with cabbage for the the family dinner, laid them on the table upon the acetite, and cut them down with a knife; by this operation both were incorporated; the whole was then put into a pot, boiled with potatoes, and chopped into a mass for dinner.

This dish was entirely eaten by the master, mistress, a daughter, her husband, and two apprentices.

After dinner the lad wished to prepare his poultice, but the materials could not be found. As a curious taste had been observed by them all in their food, they became alarmed; and on examining the table, it was evident that the sugar of lead and cabbage had been bruised together, as some in a powdered state still adhered to it.

I was sent for directly, and was soon with them; an emetic was given to five of them, which operated well, and they never found any further inconvenience; the sixth, however, one of the apprentices, as he felt nothing uneasy about him, refused to take any thing; but although the whole he had taken remained in his bowels, he never had the smallest complaint, and was as regular in his belly as before.

In Vol. III. Numb. XVI. of your Journal, you have published an extract from the Annals of Medicine, of a case of mine, where the uterus was extirpated with success; in Vol. VII. Numb. XXIX. a case is related by Mr. Watkinson, of the fatal termination of a similar operation, arising from the slipping off of the ligature, with a proposal for passing a needle through the upper part of the tumour. In the case that occurred with me, the ligature was a very strong one, and firmly applied for six hours before the operation, and no difficulty whatever took place in retaining the divided vagina till the blood vessels were secured. Were I, however, again to perform the operation, I would allow the original ligature to stay on; and without taking up any blood vessels, let the retraction take place, but leave the ends hanging out beyond the os externum for four or five days, and then pull them away if become loose.

If, however, this mode should appear uncertain, there can be no doubt but with a crooked needle every blood vessel can be secured with ease; and there never will be any necessity for employing an operation so dreadful in the eyes of every patient as the actual cautery.

My patient is still living in the neighbourhood of Glasgow, in good health. I am, &c.

*Dumbarton, Jan. 12, 1803.* ALEXANDER HUNTER.

